# Epigallocatechin-3-gallate, a prototypic chemopreventative agent for protection against cisplatin-based ototoxicity

**DOI:** 10.1038/cddis.2017.314

**Published:** 2017-07-13

**Authors:** Vikrant Borse, Raheem F H Al Aameri, Kelly Sheehan, Sandeep Sheth, Tejbeer Kaur, Debashree Mukherjea, Srinivasan Tupal, Michelle Lowy, Sumana Ghosh, Asmita Dhukhwa, Puspanjali Bhatta, Leonard P Rybak, Vickram Ramkumar

**Affiliations:** 1 Department of Pharmacology, Southern Illinois University School of Medicine, Springfield, IL 62794-9629, USA; 2 Department of Surgery (Otolaryngology), Southern Illinois University School of Medicine, Springfield, IL 62794, USA; 3 Department of Otolaryngology, Washington University School of Medicine, St Louis, MO 63110, USA

## Abstract

Cisplatin-induced ototoxicity is one of the major factors limiting cisplatin chemotherapy. Ototoxicity results from damage to outer hair cells (OHCs) and other regions of the cochlea. At the cellular level, cisplatin increases reactive oxygen species (ROS) leading to cochlear inflammation and apoptosis. Thus, ideal otoprotective drugs should target oxidative stress and inflammatory mechanisms without interfering with cisplatin's chemotherapeutic efficacy. In this study, we show that epigallocatechin-3-gallate (EGCG) is a prototypic agent exhibiting these properties of an effect otoprotective agent. Rats administered oral EGCG demonstrate reduced cisplatin-induced hearing loss, reduced loss of OHCs in the basal region of the cochlea and reduced oxidative stress and apoptotic markers. EGCG also protected against the loss of ribbon synapses associated with inner hair cells and Na^+^/K^+^ ATPase *α*1 in the stria vascularis and spiral ligament. *In vitro* studies showed that EGCG reduced cisplatin-induced ROS generation and ERK1/2 and signal transducer and activator of transcription-1 (STAT1) activity, but preserved the activity of STAT3 and Bcl-xL. The increase in STAT3/STAT1 ratio appears critical for mediating its otoprotection. EGCG did not alter cisplatin-induced apoptosis of human-derived cancer cells or cisplatin antitumor efficacy in a xenograft tumor model in mice because of its inability to rescue the downregulation of STAT3 in these cells. These data suggest that EGCG is an ideal otoprotective agent for treating cisplatin-induced hearing loss without compromising its antitumor efficacy.

Cisplatin is a platinum-based chemotherapeutic agent with proven efficacy against solid tumors. However, the clinical use of cisplatin is limited by the development of permanent hearing loss in cancer patients. There is currently no drug approved by the US Food and Drug Administration for cisplatin-induced hearing loss.^1, 2^ Studies have shown that cisplatin profoundly damages outer hair cells (OHCs) in the hook region, basal and middle turns of the cochlea^3, 4^ while sparing of inner hair cells (IHCs) in these regions. Other regions of the cochlea such as the spiral ligament (SL), stria vascularis (SV) and spiral ganglion nerve (SGN) fibers are also susceptible to cisplatin-induced damage.^5, 6, 7, 8, 9^

The generation of reactive oxygen species (ROS) has long been recognized as an important contributor to cisplatin-induced hearing loss.^10, 11, 12, 13, 14^ Antioxidants showed good promise initially for treating cisplatin-induced hearing loss, but concerns that these agents could inhibit cisplatin’s chemotherapeutic efficacy^15^ have reduced interest in developing antioxidants as otoprotectants. Recent studies have shown that cisplatin activates the mitogen-activated kinase (MAPK) pathway, linked to downstream targets such as signal transducer and activator of transcription-1 (STAT1) and p53 activation. This cascade leads to inflammation and apoptosis of OHCs and hearing loss.^16, 17, 18, 19, 20, 21^ STAT1 contributes to a drug-resistant phenotype in a number of cancers. In this study, we focused on STAT1 as a target for new drug development against cisplatin-induced hearing loss. We reasoned that inhibition of STAT1 would protect the OHCs while facilitating cisplatin-induced killing of cancer cells.

Epigallocatechin-3-gallate (EGCG) is an abundant polyphenol in green tea extract, which possesses antioxidant, anti-inflammatory and antitumorigenic properties,^22, 23, 24, 25, 26^ and also a known inhibitor of STAT1.^21, 27^ Studies have shown beneficial effects of EGCG in treating diabetes, cancer, neurodegenerative disorders, cardiovascular disease and obesity.^28, 29, 30, 31^ Our interest in EGCG stems from the fact that it provides an orally effective STAT1 inhibitor with anticancer properties, which could complement the overall therapeutic benefits of cisplatin.

## Results

### EGCG inhibits cisplatin-mediated apoptosis in UB/OC-1 cells and protects against hearing loss

**Figure 1:**
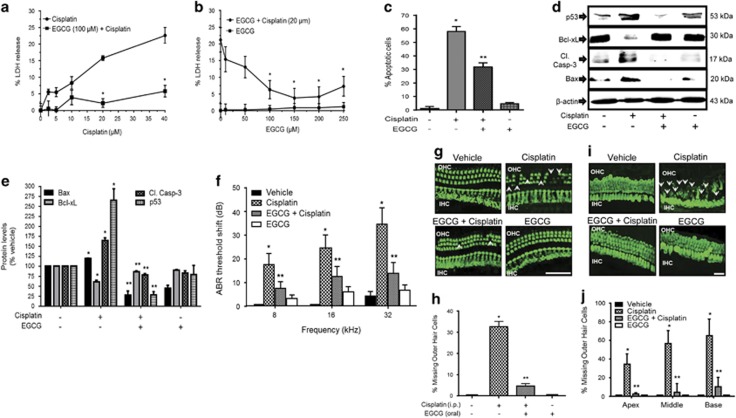
EGCG protects against cisplatin-induced apoptosis and hearing loss. (**a**) UB/OC-1 cells were pretreated with vehicle or EGCG (100 *μ*M) for 45 min, followed by different doses of cisplatin for 48 h. Cytotoxicity was assessed by LDH assays. Results are presented as the mean±S.E.M. of five independent experiments. Asterisk (*) indicates statistically significant difference (*P*<0.05) from 0 cisplatin, whereas (**) represents statistically significant difference in the EGCG+cisplatin compared with cisplatin for each cisplatin concentration. (**b**) UB/OC-1 cells were pretreated with vehicle or different doses of EGCG for 45 min, followed by 20 *μ*M cisplatin for 48 h. EGCG dose-dependently reduced the percent LDH release with statistically significant effects obtained at 50 *μ*M EGCG and maximal effects obtained at 150 *μ*M EGCG. Asterisk (*) indicates statistically significant difference compared with the no EGCG group (*P*<0.05, *n*=5). (**c**) UB/OC-1 cells were pretreated with 100 *μ*M EGCG and then cisplatin (20 *μ*M) for 48 h. Apoptosis was determined by measuring the percentage of Annexin-positive and Annexin plus propidium iodide-positive cells (lower right and upper right quadrant, respectively) by flow cytometry. Analysis of the data shows that EGCG protects against cisplatin-induced apoptosis in UB/OC-1 cells, which is plotted in cisplatin-induced apoptosis was significantly reduced by 100 *μ*M EGCG (*P*<0.05, *n*=4). (**d**) UB/OC-1 cells were pretreated with vehicle or EGCG, followed by cisplatin (as above) for 24 h. Cell lysates were used for western blot analyses of proapoptotic proteins, such as p53, Cl. Casp-3 and Bax and antiapoptotic protein, Bcl-xL. (**e**) Results from (**d**) were obtained following normalization to *β*-actin bands and are presented as the mean±S.E.M. of six independent experiments. *P<0.05 *versus* vehicle and **P<0.05 *versus* vehicle+cisplatin (*n*=4). (**f**) ABR thresholds were recorded in Wistar rats, which were then pretreated with oral EGCG (100 mg/kg body weight) on day 1. Cisplatin was administered (intraperitoneally) 24 h later and animals were continued on daily oral EGCG treatments for an additional 3 days. ABRs were performed on day 4, following which rats were killed and cochleas were processed for whole-mount studies. (**g**) Whole-mount sections were stain with Myo VIIa (green). Representative whole-mount image shows significant damage in the OHCs (white arrowheads) of basal turn of the cochlea. Scale bar is 50 *μ*m. (**h**) Percentage of missing OHCs in the basal turn of the cochlea shows significant reductions in the percent missing OHCs in EGCG treated rat cochlea compared with cisplatin-treated rat cochlea. (**i**) Cochlea explant culture were pretreated with vehicle or EGCG (100 *μ*M) for 45 min, followed by 20 *μ*M of cisplatin for 48 h. Then, explants were stained with Myo VIIa (green). Representative explant image shows significant damage in the OHCs (white arrows) of basal turn of the cochlea. Scale bar is 25 *μ*m. (**j**) Percentage of missing OHCs in the apex, middle and base of the cochlea shows significant reduction in the percent missing OHCs in EGCG-treated cochlea compared with cisplatin-treated cochlea (*P<0.05 *versus* vehicle and **P<0.05 *versus* vehicle+cisplatin, *n*=4). Cl. Casp-3, cleaved caspase-3; Myo VIIa, myosin VIIa

Cisplatin-induced cytotoxicity was measured by monitoring lactate dehydrogenase (LDH) release into the culture media. Cisplatin dose-dependently increased LDH release by 22.6±0.8% at 40 *μ*M cisplatin with a 50% of maximal effect (EC50) being ~15 *μ*M (Figure 1a). EGCG (100 *μ*M) significantly reduced LDH release elicited by cisplatin to 5.8±0.7% at 40 *μ*M cisplatin. The effect of EGCG was dose dependent, with ~41% LDH release observed at 50 *μ*M EGCG (~41% reduction), with maximal response obtained at 150 *μ*M EGCG (Figure 1b). Cisplatin also increased apoptosis of UB/OC-1 cells (66.5±3.8% of apoptotic cells), which was reduced by EGCG (31.7±2.9% of apoptotic cells) (Figure 1c). In addition cisplatin significantly increased apoptotic markers, such as p53, cleaved caspase-3 and Bax, but reduced the levels of the antiapoptotic protein, Bcl-xL. These apoptotic proteins were substantially reduced by EGCG while the reductions in Bcl-xL were attenuated (Figures 1d and e). Auditory brainstem response (ABR) studies in male Wistar rats were significantly elevated by cisplatin, with thresholds averaging 17.1±4.8, 24.3±5.5 and 34.3±6.9 dB at 8, 16 and 32 kHz, respectively. Daily orally administered EGCG protected from ABR threshold shifts at all frequencies tested (Figure 1f) and protected against loss of OHCs in the basal turn of the cochlea in whole-mount preparations (Figure 1g). Manual counts of OHCs from the basal turns indicated 32.6±2.5% loss of OHCs loss per microscope field in the cisplatin-treated group, compared with a 4.6±1.2% loss in cochleae in the EGCG plus cisplatin group (Figure 1h). No significant loss of OHCs in the middle and apical turns of cochlea induced by cisplatin was seen (data not shown), in spite of distinct hearing loss observed with cisplatin at lower to middle frequencies (Figure 1h). In explant cultures of postnatal day 3–5 cochleae cisplatin (20 *μ*M) or a combination of 100 *μ*M EGCG+cisplatin for 48 h, we observed a 65.2±7.9% loss in myosin VIIa staining in the basal turn, 56.7±7.9% loss in the middle turn and 34.4±4.9% loss in the apical turn (Figures 1i and j). Explants pre-treated with EGCG showed significantly reduced loss of myosin VIIa staining, which averaged 10.2±4.4, 4.4±4.1 and 2.4±0.6% for the basal, middle and apical turns of the cochlea (Figures 1i and j). These results suggest that in addition to intrinsic regional differences, other factors likely contribute to the limited effects of cisplatin observed in OHCs in the middle and apical turns *in vivo*.

### EGCG protected against cisplatin-mediated loss of ribbon synapses and Na^+^/K^+^ ATPase *α*1 levels in rat cochlea

**Figure 2:**
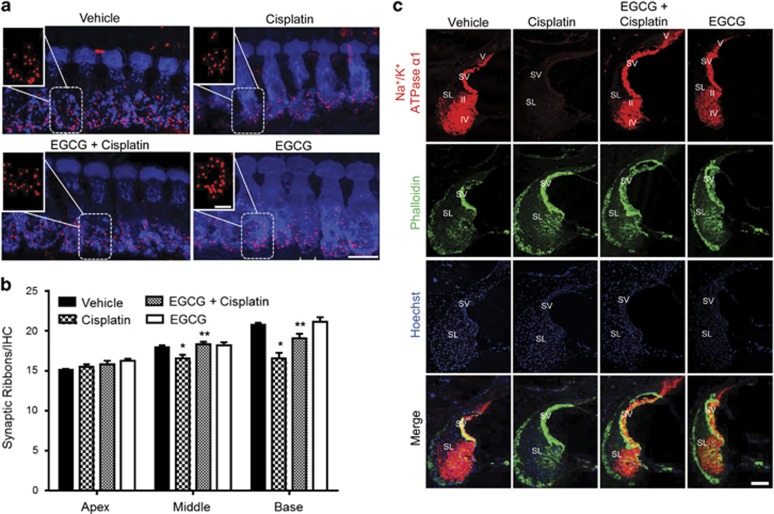
Oral EGCG inhibited cisplatin-induced loss of ribbon synapses and Na^+^/K^+^ ATPase *α*1 levels in rat cochlea. (**a**) Male Wistar rats were pretreated with oral EGCG (100 mg/kg body weight) and treated with cisplatin 24 h later. Daily oral EGCG treatments were continued for an additional 3 days. On day 4, rats were killed under anesthesia, cochleae were collected and processed for whole mounts. Whole-mount sections were stain with hair cell marker, Myo VIIa (blue), and ribbon synapse marker, CtBP2 (red). Representative whole-mount images from the basal turns show that cisplatin reduced the number of synaptic ribbons per IHC, but this effect was reduced by EGCG. Scale bar represents 10 *μ*m. (**b**) Plots are shown as synaptic ribbons per IHC in the basal, middle and apical turns of the rat cochlea. The number of ribbons in the vehicle group show a significant apex to base increase. Cisplatin decreased the number of synaptic ribbons per IHC in the base and middle turns of the cochlea but not in the apex. EGCG reduced the loss of synaptic ribbons per IHC. (**c**) Mid-modiolar sections were stained with Na^+^/K^+^ ATPase *α*1 (red), phalloidin (green) and Hoechst (blue). Cisplatin-induced reduction in the levels of Na^+^/K^+^ ATPase *α*1 were reversed by treatment with EGCG. Scale bar is 100 *μ*m (*n*=4). Myo VIIa, myosin VIIa

To address the apparent discrepancy between ABR changes and lack of OHC loss in the middle and apical turns observed *in vivo*, we assessed the integrity of IHC ribbon synapses using the presynaptic marker, C-terminal binding protein 2 (CtBP2). The average number of synaptic ribbons per IHC in vehicle control rats was 20.8±20.5, but was significantly reduced to 16.3±0.7 by cisplatin. EGCG protected against the loss of CtBP2 staining (19.0±0.6) without significantly altering staining when administered alone (Figures 2a and b). Statistically significant loss of CtBP2 staining induced by cisplatin was also observed in the middle turn but not in the apical turn of the cochlea (Figure 2b).

Using antibody against Na^+^/K^+^ ATPase *α*1 subunit,^35, 36^ we examined the levels of this enzyme in the SV and SL regions, where they regulate the endocochlear potential (EP).^32, 33, 34^ Immunolabeling of mid-modular section of rat cochlea showed that cisplatin inhibited Na^+^/K^+^ ATPase *α*1 levels in the SV and fibrocytes localized to the SL, which were attenuated in cochleae from rats treated with EGCG (Figure 2c). Cisplatin reduced the levels of Na^+^/K^+^ ATPase *α*1 in type II/IV fibrocytes, type V fibrocytes of SL and SV to 17.8±5.0%, 15.1±3.7% and 8.8±1.1% of vehicle, respectively. The reductions observed in the EGCG+cisplatin group were 81.2±24.3%, 89.5±18.1% and 72.7±15.6% of vehicle, respectively. These effects of EGCG were statistically significant (*P*<0.05, *n*=4). As Na^+^/K^+^ ATPase has a major role in maintaining the EP,^32^ we conclude that the preservation of this enzyme, in addition to ribbon synapses, by EGCG could contribute to its overall otoprotective action.

### EGCG attenuates cisplatin-induced ROS generation and downstream signaling pathway

**Figure 3:**
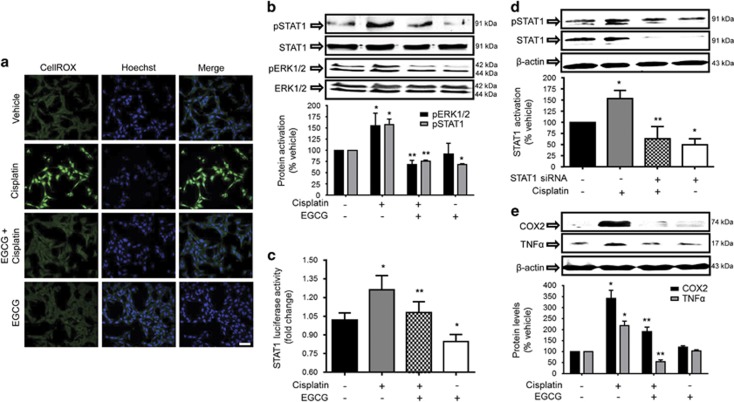
EGCG inhibits cisplatin-induced ROS activation and downstream signaling targets in UB/OC-1 cells. (**a**) UB/OC-1 cells were pretreated with vehicle or EGCG (100 *μ*M) for 45 min, followed by 2.5 *μ*M of cisplatin for 45 min. ROS generation was assessed by CellROX assays, which shows increased green fluorescence by cisplatin, but suppression of this fluorescence by EGCG. Scale bar is 50 *μ*m. (**b** and **e**) UB/OC-1 cells were pretreated with vehicle or EGCG, followed by 2.5 *μ*M of cisplatin for 24 h. Cell lysates were used for western blot analysis of phosphorylated (p)-STAT1, STAT1, p-ERK1/2, ERK1/2, COX2, TNF-*α* and *β*-actin. ERK1/2, STAT1 and *β*-actin bands (for COX2 and TNF-*α*) were used for normalization. Asterisk (*) indicates statistical significance in cisplatin *versus* vehicle groups (*P*<0.05), while (**) indicates significant difference between the cisplatin *versus* cisplatin+EGCG groups (*n*=6). (**c**) UB/OC-1 cells were co-transfected with Luciferin-coupled STAT1 and *Renilla* plasmids, pretreated with 100 *μ*M EGCG, followed by cisplatin. Lysates collected were used to perform luciferase assays, with *Renilla* luciferase used for normalization. Asterisk (*) indicates that cisplatin increased STAT1 luciferase activity, while pretreatment with EGCG significantly reduced this activity (*n*=3). (**d**) UB/OC-1 cells were transfected with scramble siRNA or STAT1 siRNA, followed by cisplatin for 45 min and lysates were used for western blot analysis to assess STAT1 phosphorylation. STAT1 and *β*-actin bands were used for normalization and results are presented as the mean±S.E.M. of four independent experiments. COX2, cyclooxygenase 2; ERK, extracellular-signal-regulated kinase 1/2; siRNA, small interfering RNA; TNF, tumor necrosis factor-*α*

Since EGCG is considered an antioxidant,^29^ we examined its efficacy to reduce cisplatin-induced ROS generation. Cisplatin (2.5 *μ*M) increased ROS generation in UB/OC-1 cultures, which was reduced by pre-treatment with 100 *μ*M EGCG for 45 min (Figure 3a). As ROS act as upstream activators of ERK1/2 and STAT1,^17^ we examined the effects of cisplatin and EGCG on these proteins. Western blotting studies showed that cisplatin increased the levels of p-ERK1/2 and p-STAT1 (Figure 3b), which were reduced in cells pretreated with EGCG. EGCG added alone significantly reduced ERK1/2 and STAT1 activity. EGCG also reduced STAT1 activity in cells transiently expressing the STAT1 luciferase plasmid (Figure 3c). STAT1 siRNA reduced both basal and cisplatin-activated STAT1 activation (Figure 3d). EGCG also inhibited STAT1-regulated gene products, such as COX2 and TNF-*α*, which were increased by cisplatin (Figure 3e). STAT1 siRNA reduced both basal and cisplatin-activated ROS generation in UB/OC-1 cells (see Supplementary Figure 1).

**Figure 4:**
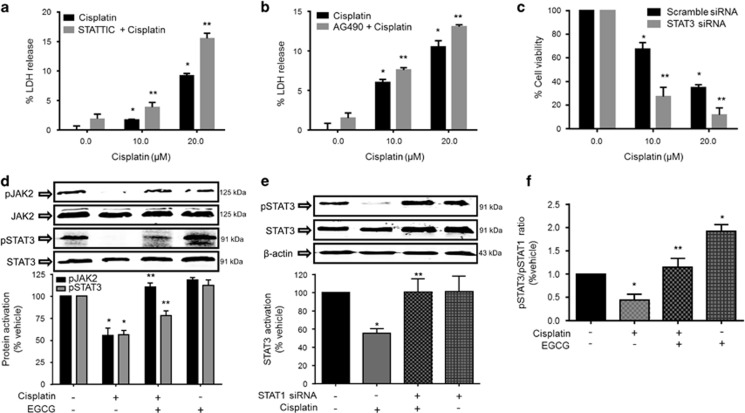
Cisplatin-induced inhibition of JAK2/STAT3 survival pathway was attenuated by EGCG in UB/OC-1 cells. UB/OC-1 cells were pretreated with (**a**) vehicle or STATTIC (100 nM) or (**b**) AG490 (10 *μ*M) for 45 min, followed by different doses of cisplatin for 48 h. Cytotoxicity, assessed by LDH assays, shows statistically significant dose-dependent increase in LDH release by cisplatin, which was enhanced by STATTIC and AG490. (**c**) UB/OC-1 cells were transfected with scramble siRNA or STAT3 siRNA, followed by different doses of cisplatin for 48 h. Cell viability assessed by MTS assays show significantly lower cell viability with knockdown of STAT3 as compared with vehicle-treated cells. For (**a–c**), results are presented as the mean±S.E.M. of three independent experiments. Asterisk (*) indicates statistically significant difference from vehicle-treated cells (no cisplatin), while (**) indicates significant difference from cisplatin-treated group (*P*<0.05). (**d**) UB/OC-1 cells were pretreated with vehicle or EGCG (100 *μ*M) for 45 min, followed by 20 *μ*M of cisplatin for 24 h. Cell lysates were used for western blot analysis of phosphorylated (p)-JAK2, JAK2, p-STAT3 and STAT3. JAK2 and STAT3 bands were used for normalization and results are presented as the mean±S.E.M. of four independent experiments. (**e**) UB/OC-1 cells were transfected with scrambled siRNA or STAT1 siRNA, followed by 24 h treatment with cisplatin. Western blot analyses were performed for p-STAT3, STAT3 and *β*-actin. STAT3 and *β*-actin bands were used for normalization and results are presented as the mean±S.E.M. of four independent experiments. (**f**) Plot of p-STAT3/p-STAT1 ratios based on data shown in Figures 3b and 4e. Asterisk (*) indicates statistical significance *versus* vehicle controls, while (**) indicates statistical significance compared with cisplatin-treated groups (*P*<0.05, *n*=4). JAK2, Janus kinase 2; siRNA, small interfering RNA

Previous studies showed that cisplatin inhibited p-STAT3,^37, 38^ which is important for cell survival.^39^ Inhibition of STAT3 activation in UB/OC-1 cells with STATTIC (100 nM) (73.1±4.5% inhibition) enhanced cisplatin cytotoxicity (Figure 4a). Furthermore, inhibition of JAK2 activation, the immediate regulator of p-STAT3 levels using AG490 (10 *μ*M) (Figure 4b) or knockdown of STAT3 by siRNA (by >70%) enhanced killing of UB/OC-1 cells by cisplatin (Figure 4c). These data support a protective role of the JAK2/STAT3 signaling pathway in UB/OC-1 cells and suggest that maintenance of this pathway is essential for cell survival. Western blots showed that cisplatin significantly inhibited pJAK2 and p-STAT3 levels in UB/OC-1 cells, whereas EGCG pre-treatment attenuated these responses (Figure 4d). Knockdown of STAT3 increased basal but not cisplatin-induced ROS generation (Supplementary Figure 1). To determine whether the suppression of p-STAT3 by cisplatin was a result of its activation of STAT1, we knocked down STAT1 by siRNA and showed that this significantly attenuated cisplatin-induced decrease in STAT3 phosphorylation (Figure 4e). The ratio of the cellular p-STAT3/p-STAT1 in the presence of cisplatin was significantly decreased to 0.44±0.10, but was relatively unchanged at 1.14±0.18 in the EGCG+cisplatin group. Addition of EGCG alone produced an additional increase in this ratio to 1.92±0.14 (Figure 4f).

### EGCG-mediated inhibition of cisplatin-induced apoptosis involves inhibition of ERK1/2, STAT1 activation and downstream inflammatory markers in rat cochlea

**Figure 5:**
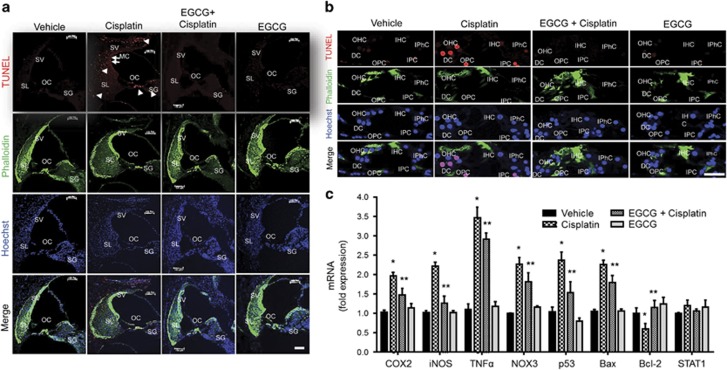
EGCG inhibits cisplatin-mediated apoptosis and inflammatory response in rat cochlea. (**a**) Male Wistar rats were pretreated with oral EGCG (100 mg/kg body weight) for 24 h, followed by cisplatin (11 mg/kg) and daily oral EGCG treatments were continued for an additional 3 days and killed on day 4. Cochleas were collected, fixed, decalcified and processed for mid-modiolar sections. Sections were used for TUNEL staining (red) along with phalloidin immunostaining (green). Cisplatin increased TUNEL-positive nuclei (red) in the OC, SL, SV and SGN, which was blocked by pre-treatment with oral EGCG. Scale bar represents 100 *μ*m. (**b**) In the OC region, TUNEL-positive nuclei (red) was observed in the OHCs, DCs, IPCs and OPCs of cisplatin-treated cochlea. No distinct TUNEL-positive nuclei were observed in IHCs and IPhCs. Oral EGCG inhibited the TUNEL-positive response induced by cisplatin in the OC. Scale bar represents 25 *μ*m. (**c**) Cochleae were collected for real-time RT-PCR to determine the levels of *COX2*, *iNOS*,*TNF-α*,*NOX3*, *p53*, *Bax*, *Bcl-2* and *STAT1* mRNAs. The figures in (**a**) and (**b**) are representatives of cochleas from different groups of four rats each. Data presented in (**c**) represent the mean±S.E.M. of cochleas from four animals. Asterisk (*) indicates statistically significant difference from vehicle, while (**) indicates significant difference from the cisplatin group (*P*<0.05)

In mid-modiolar sections we show that cisplatin increased TUNEL-positive cells in the organ of Corti (OC), SL, SGNs and marginal cells of SV (Figure 5a). In the OC, cisplatin increased TUNEL-positive nuclei in the OHCs, DCs (Deiters cells), inner pillar cells (IPCs) and outer pillar cells (OPCs). TUNEL-positive staining was absent in IHCs and inner phalangeal cells (IPhCs) in cochleae from cisplatin-treated animals (Figure 5b). No TUNEL-positive staining was observed in cochleae from rats treated with oral EGCG. Cisplatin increased the expression of apoptotic genes, such as *p53* and *Bax*, and decreased the expression of the antiapoptotic gene *Bcl-2* in the cochleae (Figure 5c). In addition, cisplatin significantly increased the expression of cochlear inflammatory genes, such *TNFα*, *COX2*, *iNOS* and *NOX3*, which were attenuated by EGCG (Figure 5c). No significant change in *STAT1* was observed.

**Figure 6:**
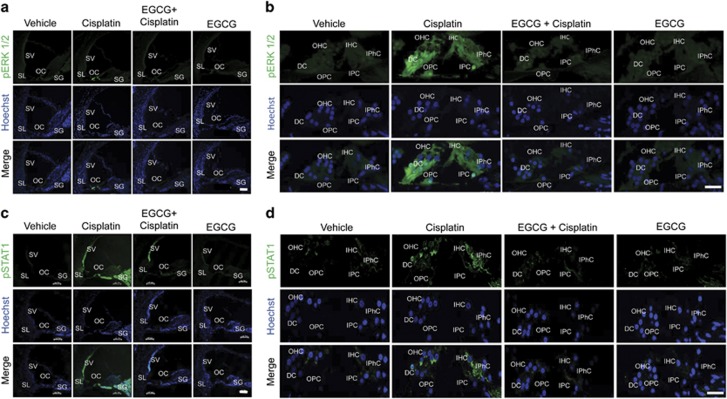
EGCG inhibits cisplatin-induced ERK1/2 and STAT1 activation in rat cochlea. Male Wistar rats were pretreated with oral EGCG (100 mg/kg body weight), followed by cisplatin (11 mg/kg) 24 h later. Daily oral EGCG treatments were continued for an additional 3 days and rats killed on day 4. Phosphorylated (p)-ERK1/2 (**a** and **b**) and p-STAT1 (**c** and **d**) immunolabeling are indicated by green fluorescence, while cell nuclei are defined by Hoechst staining. Cisplatin-induced p-ERK1/2 immunoreactivity was more localized to the OC (**a**). Higher magnification images show increased p-ERK1/2 immunolabeling in OHCs, IHCs, DCs, IPhCs, IPCs and OPCs (**b**). Cisplatin increased p-STAT1 in the OC, SL, SV and SGN, which was reduced by pre-treatment with EGCG (**c**). In the OC, cisplatin increased p-STAT1 in OHCs, IHCs, DCs and IPhCs. Scale bar for panels a and c represent 100 *μ*m, while scale bar for b and d represent 25 *μ*m. The figures are representatives of cochleas from different groups of four rats each. ERK1/2, ERK, extracellular-signal-regulated kinase 1/2

Mid-modiolar sections also revealed that cisplatin-increased p-ERK1/2 staining was localized to the OCs (Figure 6a), with immunolabeling observed in OHCs, IHCs, DCs, IPhC, IPCs and OPCs (Figure 6b). In rats pre-treated with EGCG, there was no substantial ERK1/2 activation in the OC by cisplatin (Figure 6a). Cisplatin-induced p-STAT1 immunoreactivity in OHCs, IHCs, DCs and IPhCs was attenuated by EGCG (Figures 6c and d).

### EGCG does not interfere with cisplatin anticancer effect in cancer cells

**Figure 7:**
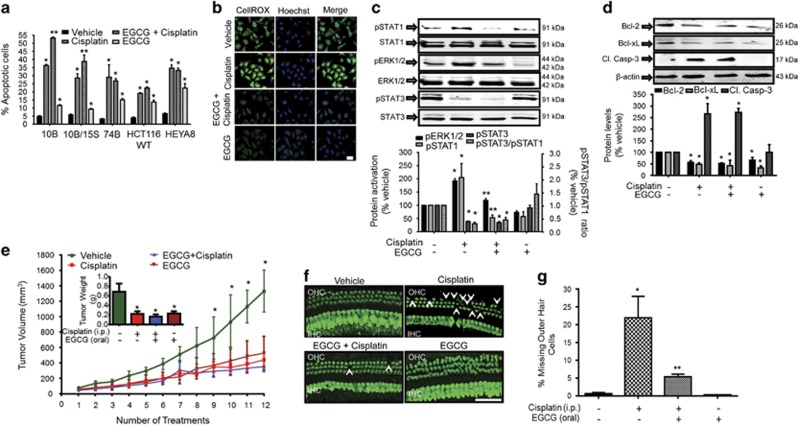
EGCG protects against cisplatin-induced OHC damage without compromising cisplatin antitumor efficacy. (**a**) All cancer cells were pretreated with 100 *μ*M EGCG for 45 min before cisplatin (10 *μ*M) for 24 h, except 10B/15 S (UMSCC 10B/15 S), which was treated for 48 h. Annexin V apoptotic assay was performed and apoptosis was quantified by flow cytometry. Cisplatin significantly increased apoptosis in all cell lines tested, which was further increased by EGCG in 10B (UMSCC 10B) and 10B/15 S cells, but not in 74B (UMSCC 74B), HCT116 WT and HEYA8 cells. EGCG alone increased apoptosis in all cell lines tested. (**b**) UMSCC 10B cells were pretreated with vehicle or EGCG, followed by 2.5 *μ*M of cisplatin for 45 min and ROS generation was assessed and imaged by a confocal microscope. Cisplatin increased ROS production, which was inhibited by EGCG. Images represent replicates of at least three different experiments. Scale bar is 25 *μ*m. (**c** and **d**) UMSCC 10B cells were pretreated with vehicle or EGCG for 45 min, followed by 10 *μ*M of cisplatin for 24 or 48 h. Cell lysates were used for western blot analysis of phosphorylated (p)-ERK1/2, p-STAT1, p-STAT3, Bcl-2 and Bcl-xL (24 h) or Cl. Casp-3 (48 h). ERK1/2, STAT1, STAT3 and *β*-actin bands were used for normalization, p-STAT3/p-STAT1 ratio was calculated and results are presented as the mean±S.E.M. of four to six independent experiments (*P<0.05 *versus* vehicle and **P<0.05 *versus* vehicle+cisplatin). (**e**) UMSCC 10B cells (1.5 × 10^6^) were subcutaneously injected in one flank of SCID mice. Mice (6 per group) were treated three times a week with vehicle, cisplatin (2 mg/kg) (intraperitoneally), oral EGCG (100 mg/kg) or oral EGCG+cisplatin. Tumor volumes were calculated based on the formula: volume=width^2^ x (length/2). Mice were killed 2 days after the last treatment (denoted as the twelveth treatment) and tumors were collected, measured for their size and weighed. All three treatment groups show significant reductions in tumor volumes as compared with vehicle after the ninth treatment (*P<0.05), which was maintained up to the twelveth treatment. There was no significant difference among the cisplatin, EGCG or EGCG+cisplatin groups. Cisplatin reduced tumor weights, which was not significantly different among the drug treatment groups. (**f**) Whole-mount preparations of cochleas stained with Myo VIIa (green) indicate loss of OHCs in the basal turn, which was reduced in the mice treated with cisplatin and EGCG. Damage to OHCs (white arrows) is represented by white arrows. Scale bar represents 50 *μ*m. (**g**) Percentage of missing OHCs in the basal turn of the cochlea is plotted in the various treatment groups (*P<0.05 *versus* vehicle (no drug treatment) and **P<0.05 *versus* vehicle+cisplatin, *n*=6). Cl. Casp-3, cleaved caspase-3; ERK1/2, ERK, extracellular-signal-regulated kinase 1/2; Myo VIIa, myosin VIIs; SCID, severe combined immunodeficiency; WT, wild type

To determine whether EGCG could alter the efficacy of cisplatin in cancers, we screened several relevant cancer cells against which cisplatin is used clinically. These include head and neck cancer cell lines (University of Michigan squamous cell carcinoma 10B (UMSCC 10B), UMSCC 74B and UMSCC 10B/15 S), colon cancer cells (HCT116 WT) and ovarian cancer cells (HEYA8). Annexin V-FITC assays showed that cisplatin induced apoptosis in UMSCC 10B and UMSCC 10B/15 S cells by 36.2±2.1% and 28.6±5.0%, respectively. However, EGCG pre-treatment significantly increased the extent of apoptosis in these cells by 53.2±1.9% and 38.9±7.8%, respectively, suggesting that EGCG could sensitize the resistant cells (UMSCC 10B/15 S) to cisplatin. EGCG pre-treatment did not alter the extent of cisplatin-induced apoptosis in UMSSC 74B, HCT116 WT and HEYA8. Interestingly, EGCG alone significantly killed head and neck cancer cells (UMSCC 10B, UMSCC 74B and UMSCC 10B/15 S), colon cancer cells (HCT116 WT) and ovarian cancer cells (HEYA8) (Figure 7a). These data suggest that EGCG did not reduce cisplatin’s anticancer property. CellROX ROS assays showed that EGCG inhibited both basal and cisplatin-induced ROS generation (Figure 7b). Similarly, EGCG reduced ROS-dependent processes, such as ERK1/2 and STAT1 activation, but not cisplatin-mediated reduction in p-STAT3 in UMSCC 10B cancer cells or the p-STAT3/p-STAT1 ratios (Figure 7c), which averaged 0.29±0.06, 0.44±0.10 and 1.42±0.39 for the cisplatin, EGCG+cisplatin and EGCG- alone groups, respectively. EGCG and cisplatin alone, or in combination, reduced the levels of Bcl-xL and Bcl-2 in UMSCC 10B cancer cells (Figure 7d). We also observed increases in cleaved caspase-3 in cisplatin and cisplatin+EGCG treatment groups after 48 h in these cells, but no apparent effect of EGCG alone on this protein (Figure 7d).

Cisplatin’s antitumor activity was examined using a xenograft model of head and neck cancer tumor cells in SCID mice. Tumors were produced by subcutaneous injections of UMSCC 10B cancer cells and cisplatin, and EGCG treatments were administered as described in Materials and Methods. We observed significant increases in tumor volumes over the treatment period, which reached an average of 1131±199 mm^3^ in vehicle-treated group at the end of the study (Figure 7e). Cisplatin significantly reduced tumor volume to 390±69 mm^3^, compared with vehicle-treated mice. Furthermore, the combination of oral EGCG with cisplatin led to a further reduction in tumor volumes to 337±76 mm^3^. The excised tumors showed statistically significant reductions in weights, compared with vehicle-treated controls (*P*<0.05, *n*=6) (Figure 7e). Cochlear OHC integrity in these mice using myosin VIIa immunostaining (Figure 7f) showed loss of OHCs in the cisplatin-treated cochleae (arrowheads), but substantial reductions in OHC loss in the EGCG+cisplatin group. IHCs appeared mostly intact in the cisplatin-treated cochleae. Quantification of myosin VIIa-labeled hair cells showed a 26.5±5.4% loss of OHCs by cisplatin in the basal turn of the cochlea, but only a 5.4±0.7% loss with EGCG+cisplatin (Figure 7g). There was no significant loss of OHCs in middle and apical turns of cochlea in SCID mice in the cisplatin-treated group (data not shown).

## Discussion

This study provides strong evidence supporting the benefits of using EGCG to treat cisplatin ototoxicity. We show that EGCG protects against cell damage and apoptosis in multiple regions of the cochlea, such as OHCs, SV and SL. Protection was also observed at the level of IHC ribbon synapses, whose levels were decreased by cisplatin. EGCG also reduced cisplatin-induced loss of Na^+^/K^+^ ATPase *α*1 subunit in type II, IV and V fibrocytes located in the SV and SL. *In vitro* studies show that EGCG possesses antioxidant, anti-inflammatory and antiapoptotic properties. Importantly, EGCG appears to be safe to use with concurrent cisplatin administration for treating cancer, as it did not interfere with cisplatin’s antitumor efficacy against head and neck cancer cells. Thus, oral EGCG could serve as an effective otoprotective drug for hearing loss.

An earlier study showed that EGCG could protect utricular cells against cisplatin-induced death *in vitro* by inhibiting the STAT1 transcription factor.^21^ We demonstrated that knockdown of STAT1 by transtympanic application of siRNA protected against cisplatin ototoxicity.^17^ STAT1 also contributed to the transient hearing loss produced by transtympanic capsaicin administration^20^ and inhibition of the STAT1 inflammatory pathway was linked to A1 adenosine receptor-mediated otoprotection.^16^ These studies support the overall goal of targeting STAT1 for treating hearing loss.

This study assessed the efficacy and mechanism of action of EGCG to treat cisplatin-induced ototoxicity. We show that EGCG inhibits cisplatin-induced STAT1 activation and STAT1-regulated genes in the cochlea. Moreover, EGCG reduced cisplatin-induced apoptotic pathway, as evidenced by reduced cell apoptosis *in vitro* and *in vivo*. Furthermore, we show that activated STAT1 negatively regulates p-STAT3, a transcription factor linked to cell survival and autophagy.^40, 41^ *In vitro* studies also show that cisplatin could reduce p-STAT3 levels by inhibiting pJAK2, which was attenuated by EGCG. Similar findings showing inhibition of STAT3 have been reported previously in the mouse cochlea.^38^ We propose that EGCG-mediated otoprotection inhibition of inflammatory and apoptotic pathways through STAT1 and preserving STAT3. The resulting net increase in the p-STAT3/p-STAT1 ratio in the cochlea would promote cell survival,^39, 40, 41^ even in presence of cisplatin.

Otorotection mediated by EGCG likely relates, in part, to its antioxidant property, as cisplatin-induced ROS generation is implicated in ototoxicity.^42^ ROS promote alteration in structural proteins and enzymes, lipid peroxidation, leading to membrane damage and DNA strand breaks, which are reduced by antioxidants.^42^ Additionally, STAT1 couples ROS to inflammatory and apoptotic pathways^17, 20^ by activating the MAPK signaling.^16, 17^ In this study, we demonstrate that cisplatin activates ERK1/2 mainly in the OC, but not in SV, SL and SGN, suggesting a more limited contribution of ERK1/2 in apoptosis of OHCs, DCs, IPCs and IPhCs and OPCs. Other MAPKs, such as p38 and c-Jun N-terminal kinases, could mediate cisplatin-induced apoptosis in SV, SL and SGN.^19^ In the OC, TUNEL-positive staining was observed in both OHCs and their supporting cells, as described previously,^3, 4, 43, 44^ which were protected by EGCG.

Functionally, EGCG protected against cisplatin-induced hearing loss at all frequencies tested, even though loss of OHCs was restricted to the basal region of the cochlea. Thus, targets other than OHCs in the middle and apical turns of the cochlea could contribute to lower frequency hearing loss. In explant cultures, damage to OHCs was present throughout the length of the cochlea and partly independent of frequency regions. This suggests that regional differences in OHC loss could be explained, at least in part, by the basal to apical distribution of cisplatin in the cochlea. Cisplatin also decreased the level CtBP2, a major component of ribbon synapses, in the basal and middle turns of the cochlea. These ribbon synapses are intimately involved in IHC neurotransmission, as they regulate vesicular release of neurotransmitters.^45^ Similar findings have been observed with aminoglycoside and noise.^46, 47, 48^ Based on the importance of CtBP2 and other ribbon synaptic proteins for neurotransmission in IHCs, we believe that cisplatin-induced decrease in CtBP2 could significantly impact hearing. The ability of EGCG to maintain the levels of CtBP2 staining could contribute to its overall efficacy as an otoprotective agent.

Strial Na^+^/K^+^ ATPases maintain the ionic composition of endolymph and the EP.^49, 50, 51, 52^ In particular, Na^+^/K^+^ ATPase *α*1 is present in the lateral wall of cochlea, mainly in SV and type II, IV and V fibrocytes of SL.^32, 33, 34^ Fibrocytes derived from SL also contribute to cochlear inflammation.^35, 53, 54, 55^ We found that cisplatin induced loss of Na^+^/K^+^ ATPase *α*1 expression in SV and type II, IV and V fibrocytes of SL throughout the cochlea, which was reduced by oral EGCG. The mechanism underlying the loss of Na^+^/K^+^ ATPase *α*1 levels and protection by EGCG could involve ROS-stimulated endocytosis of plasma membrane-localized enzyme.^56^

**Figure 8:**
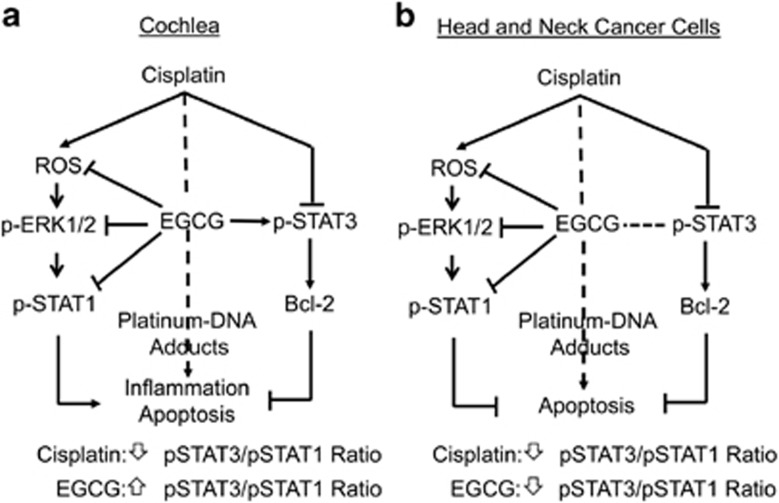
Proposed mechanism of action of cisplatin and EGCG in cochlea and cancer cells. (**a**) Cisplatin activates STAT1 through a ROS/MAPK pathway and reduces phosphorylated (p)-STAT3. The reduction in p-STAT3/p-STAT1 ration promotes inflammation and apoptosis in the cochlea. EGCG promotes cell survival in the cochlea by inhibiting STAT1 and protecting p-STAT3, thereby increasing the p-STAT3/p-STAT1 ratio and regulating the expression Bcl-2 and other prosurvival factors. (**b**) Cisplatin kills cancer cells primarily by forming platinum–DNA adducts, which inhibits DNA replication. Cisplatin-induced activation of STAT1 (which contributes to survival and drug resistance) and inhibition of STAT3 (contributes a survival signal). The STAT3/STAT1 ratio and promotes apoptosis. EGCG antitumor contribution is likely produced, in part, through inhibition of p-STAT1 without affecting p-STAT3. The combination of EGCG with cisplatin leads to suppression of both STAT1 and STAT3 pathways, leading to greater cell apoptosis. MAPK, mitogen-activated protein kinase

Our study showed that EGCG did not interfere with cisplatin's chemotherapeutic efficacy against head and neck tumors in SCID mice and did not affect cisplatin-induced killing of cancer cells *in vitro*. *In vitro* studies showed that EGCG inhibited cisplatin-induced ROS, ERK1/2 and STAT1 activation in UMSCC head and neck cancer cells, but had little effect on cisplatin-induced inhibition of STAT3, Bcl-2 and Bcl-xL levels or the observed increases in cleaved caspase-3. Increases in ROS, ERK1/2 and STAT1 confer resistance and poor survival outcomes in patients on cisplatin.^57^ However, STAT3, Bcl-2 and Bcl-xL are crucial for the survival of cancer cells and thus inhibition by both cisplatin and (possibly EGCG) could account for the lack of anticancer interference with EGCG. EGCG has been shown to significantly downregulate STAT1 and STAT3 expression in CAL-27 human squamous carcinoma cells.^58^ STAT1 is upregulated in certain drug-resistant cancers and inhibition of STAT1 by EGCG in colorectal cancer cells could contribute to the antitumor actions of this drug.^59^ Studies in SCID mice support the *in vitro* finding of lack interference of cisplatin antitumor efficacy by EGCG. The SCID mouse model allowed for simultaneous examination of cochlear damage and tumor growth. Using this model, we clearly demonstrate that EGCG did not interfere with cisplatin’s antitumor efficacy even though it suppressed damage to OHCs. An important insight gleaned from this study is that important differences in molecular signaling pathways exist between normal cochlear cells and head and neck cancer cells, which could be exploited by drugs, such as EGCG, to provide effective otoprotection without compromising antitumor efficacy. Specifically, EGCG differently regulates the levels of STAT1, STAT3 and Bcl-xL proteins in normal cochlear and cancer cells (see Figure 8). The ability of EGCG to restore the levels of p-STAT3 and Bcl-xL in the cochlear cells, but not in cancer cells, could account for its protection of the cochlea without compromising cisplatin’s chemotherapeutic efficacy. This finding provides an additional advantage of coadministering EGCG along with cisplatin in the clinic for treating cancers and could permit increasing the dose of cisplatin to treat drug-resistant tumors.

The physiologically relevant serum concentrations of EGCG are in the high nanomolar range^60^ and contrasts the high concentrations of the drug used *in vitro* to show cytoprotection in these and other studies.^61^ Oral consumption of EGCG at a dose comparable to that used in this study resulted in serum levels in the submicromolar range,^62^ suggesting that otoprotection was achieved at submicromolar concentrations of EGCG in the serum. Using a formula for dose translation from animal to human, we calculated an equivalent dose of EGCG in human to be 32 mg/kg (compared with the animal dose used in this study) for an individual weighing 60 kg.^63^ The similarities in responses observed with *in vitro versus in vivo* EGCG suggest that similar mechanisms mediate these responses and indicate that the *in vitro* model is relevant to the rodent model of cisplatin ototoxicity.

EGCG shows promise for the treatment of metabolic disorders, cardiovascular diseases, neurodegenerative diseases and cancers.^23, 28, 30, 31, 64, 65^ A number of clinical trials are currently in progress to study the effect of EGCG in various cancer types and other diseases. These trials have shown chemopreventative effects of EGCG, in cervical cancer,^66^ metachronous colorectal adenomas^67, 68^ and prostate cancer.^69, 70^ These studies make it more feasible that EGCG would be advanced into clinical trials for treating hearing loss in patients with cisplatin-based therapy for treatment of cancer.

Overall, this study provides strong support for the use of EGCG as an adjunctive therapy along with cisplatin for the treatment of solid tumors. The use of EGCG should substantially decrease the dose-limiting side effects of cisplatin, namely ototoxicity and nephrotoxicity, and allow for use of higher drug concentrations to treat cisplatin-resistant tumors.

## Materials and methods

### Drugs and reagents

Cisplatin, EGCG, poly-l-ornithine 0.01% solution, STATTIC, AG490, laminin and phosphatase inhibitor cocktails 2 and 3 were purchased from Sigma-Aldrich (St. Louis, MO, USA).

### Antibodies

Various primary antibodies (with catalog numbers and companies) used were as follows: p-ERK1/2 (no. 7976), ERK1/2 (no. 93), STAT1 (no. 592), Na+/K+ ATPase *α*1 (no. 21712) STAT3 (no. 8019), Bcl-xL (no. 8392), *β*-actin (no. 69879) from Santa Cruz Biotechnology (Dallas, TX, USA); JAK2 (no. 3230), pJAK2 (no. 3776), p-STAT3 Tyr705 (no. 4113 S), p-STAT1 Ser727 (no. 9177 S), p53 (no. 2524), Bcl-2 (no. 2876), cleaved caspase-3 (no. 9661), Bax (no. 2772), TNF*α* (no. 3707 S), Cox2 (no. 4842) from Cell Signaling Technology (Danvers, MA, USA); myosin VIIa rabbit polyclonal (no. 25-6790) from Proteus Biosciences (Ramona, CA, USA); CtBP2 mouse IgG1 (no. 612044) from BD Biosciences (San Jose, CA, USA); Alexa Fluor 488 Phalloidin (no. A-12379) from Thermo Fisher Scientific (Waltham, MA, USA). Secondary antibodies used were as follows: donkey anti-rabbit IRDye 680RD (no. 926-68073), donkey anti-goat IRDye 800RD (no. 926-32212), goat anti-mouse IRDye 800RD (no. 926-32214) from LI-COR Biosciences (Lincoln, NE, USA); Alexa Fluor 488 goat anti-rabbit (no. A11008) from Thermo Fisher Scientific; Alexa Fluor 647 goat anti-mouse IgG1 (no. A-21240) from Molecular Probes (Eugene, OR, USA).

### Cell culture

Immortalized OC cells derived from the mouse, UB/OC-1 cells, were obtained from Dr. Matthew Holley (Institute of Molecular Physiology, Addison Building, Western Bank, Shefield, UK) and cultured in RPMI-1640 media (HyClone, Logan, UT, USA) supplemented with 10% Fetalclone II serum (Hyclone), penicillin–streptomycin (Invitrogen, Carlsbad, CA, USA) and normocin (InvivoGen, San Diego, CA, USA). Cultures were grown at 33 °C in a humidified incubator with 10% CO_2_. Cells were passaged two times a week and all the experiments were performed using subconfluent monolayers. Head and neck cancer cell lines, UMSCC 10B, UMSCC 74B (cisplatin-sensitive) and UMSCC 10B/15 S (cisplatin-resistant) were provided to us by Dr. Krishna Rao, Southern Illinois University (SIU) School of Medicine (Springfield, IL, USA).^71^ Ovarian cancer cell line (HEYA8) and colon cancer cell line (HCT116 WT) were provided by Dr. Daotai Nie and Dr Subhas Chakrabarty, respectively (SIU, School of Medicine, Springfield, IL, USA). All cancer cell lines were cultured in DMEM (HyClone) supplemented with 10% fetal bovine serum (Atlanta Biologicals Inc., Flowery Branch, GA, USA) and penicillin–streptomycin (Invitrogen).

### Animal procedures and sample collection

Adult male Wistar rats (from Envigo (Indianapolis, IN, USA), 200–250 g) were given free access to commercial food and water and were housed in temperature-controlled rooms with a 12 h light/dark cycle. Pre-treatment ABRs were performed immediately before the administration of EGCG (100 mg/kg), which was orally administered every day for 4 days. A single dose of cisplatin (11 mg/kg) was administered intraperitoneally a day after the first EGCG treatment in these animals, and then anesthetized with a mixture of ketamine and xylazine (3:1). Post-treatment ABRs were performed 72 h following cisplatin administration, after which the animals were decapitated and the cochleae were harvested. The cochleae were rapidly frozen in liquid nitrogen for total RNA or perfused with 4% paraformaldehyde for immunohistochemistry.

SCID mice (from Envigo, 5–6 weeks of age) were subcutaneously injected with 1.5 × 10^6^ mycoplasma-free UMSCC 10B cells in one flank. When tumors reached palpable size (~100 mm^3^), attained in 10–15 days after injections, mice were pre-treated with oral PBS (vehicle) or oral EGCG (100 mg/kg), and each group were then treated with intraperitoneal PBS or intraperitoneal cisplatin (2 mg/kg). Animals were subsequently treated with oral EGCG, followed by intraperitoneal cisplatin, on alternate days three times per week for a total of 11 treatments. At the time of each treatment, tumor volumes were calculated based on the formula: volume=width^2^ x (length/2).^72^ Mice were killed 24 h after the eleventh treatment. All animal procedures were approved and monitored by the SIU, Laboratory Animal Care and Use Committee.

### Evoked potentials

ABRs were determined as described previously.^16^ Animals were anesthetized and moved to a double-walled radio frequency shielded sound proof booth and secured with hollow ear bars. Subdermal electrodes were positioned at the vertex of the skull (active), in the hind flank muscle region (ground) and under the pinna of the ear to detect the response. Ear phones were placed into the ears to provide sound stimulus. Animals were tested with a stimulus intensity series that was initiated at 10 dB SPL and reached a maximum at 90 dB SPL, with 10 dB increments. The auditory stimuli included tone bursts at 8, 16 and 32 kHz with a 5 ms plateau and a 1 ms rise/fall time presented at a rate of 5/s. Threshold was defined as the lowest intensity capable of evoking a reproducible, visually detectable response with two distinct waveforms (waves 2 and 3) and minimum amplitude of 0.5 *μ*V.

### Morphological studies by cochlear whole-mount preparation

Isolated adult rat and SCID mice cochleae were perfused with 4% paraformaldehyde and kept overnight at 4 °C in the same solution for fixation. Cochleae were decalcified in 0.1 M EDTA (pH 7.4) with stirring at room temperature for 2 weeks, followed by microdissection into basal, middle and apical turns for immunolabeling.

### TUNEL assay in cochlear sections

*In vivo* apoptosis was detected by TUNEL assay using ApopTag Red *In Situ* Apoptosis Detection Kit (EMD Millipore, Billerica, MA, USA), according to the manufacturer’s instructions. Slides were counterstained with Alexa Fluor 488 phalloidin conjugate antibody, followed by Hoechst for visualization of cell nuclei. Images were captured using a Leica Laser Scanning Confocal Microscope (Leica Microsystems Inc., Buffalo Grove, IL, USA) and analysed using the Leica Software version LAS-AF-Lite-2.6.0-266 (Leica Microsystems Inc.).

### Immunohistochemistry

Immunohistochemistry was performed as described previously.^16^ Immunostaining was initiated by blocking the cochlear sections or whole-mount preparations for 2 h in PBS containing 5% horse serum. Sections were then incubated overnight at 4 °C in primary antibody prepared in 0.2% Triton X-100 in PBS, after which they were washed three times for 5 min with PBS. They were then incubated in secondary antibodies prepared in PBS for 3 h in dark at room temperature. Sections were then stained with Hoechst and imaged using Leica Laser Scanning Confocal Microscope.

### Cochlear organotypic cultures

Cochleae were extracted from C57BL/6 neonatal mice (postnatal days 3–5) in dissection media containing 1 × Hank’s balanced salt solution and 25 mM HEPES (pH 7.5). OC explants were isolated from these cochleae and transferred to a six-well plate with coverslip precoated with 1:1 poly-l-ornithine and laminin, supplemented with 20% fetal bovine serum, containing explant culture media (DMEM with glucose and glutamine+1% fetal calf serum+ampicillin and ciprofloxacin). The explants were cultured at 37 °C in 5% CO_2_ for 1 day before drug treatments.

### Hair cell and ribbon synapse counts

Cochlear whole-mount samples were imaged by confocal microscopy. All microscope settings were constant for all samples for hair cells (myosin VIIa) count and ribbon synapses (CtBP2) count. In each region, counting was performed manually (~160 *μ*m length each) for OHCs and IHC ribbon synapses. Three random microscopic fields per sample were used for counting. Each image presented a total of 60–70 OHCs and 18–22 IHCs. Counts were normalized to those of vehicle controls. Ribbon synapses were counted per IHC and the data were presented as a number of ribbon synapses per IHC.

### RNA isolation

Cochleae were pared down to the bone, crushed in liquid nitrogen, followed by extraction in 500 *μ*l of TRI reagent. Chloroform (100 *μ*l) was added and the tube was shaken vigorously for 15 s and centrifuged at 12 000 × *g* for 15 min. RNA was extracted by washing the pellet with 0.5 ml ice-cold isopropanol, followed by cold 75% treated ethanol. The ethanol was removed and the tube was air dried briefly. The RNA pellet was resuspended in nuclease free water and RNA levels were determined using optical density readings corresponding to wavelengths of 260, 280 and 320nm using a spectrophotometer (NanoDrop ND-1000 from Thermo Fisher Scientific, Waltham, MA, USA).

### Real-time RT-PCR

Total RNA (500ng) was converted to cDNA using iScript cDNA Synthesis Kit (Bio-Rad, Hercules, CA, USA). The reaction mixture was setup as follows: 1ng of total RNA, 4 *μ*l of iScript reaction mix, 1 *μ*l of iScript reverse transcriptase and nuclease free water to bring the total volume to 20 *μ*l. The reaction mix was incubated at 25 °C for 5 min, 42 °C for 30 min and 85 °C for 5 min. This cDNA reaction mix was used for real-time PCR, as described previously.^73^ Gene-specific primer pairs were used for the various reactions and mRNA expression levels were normalized to the levels of *GAPDH*. The primer sets were purchased from Sigma Genosys (St. Louis, MO, USA), and were as follows: Rodent-GAPDH (sense): 5′-ATGGTGAAGGTCGGTGTGAAC-3′, (antisense): 5′-TGTAGTTGAGGTCAATGAAGG-3′ Rodent NOX3 (sense): 5′-GTGAACAAGGGAAGGCTCAT-3′, (antisense): 5′-GACCCACAGAAGAACACGC-3′ Rodent-TNF-*α* (sense): 5′-CAGACCCTCACACTCAGATCA-3′, (antisense): 5′-TGAAGAGAACCTGGGAGTAGA-3′ Rodent-COX2 (sense): 5′-TGATCGAAGACTACGTGCAAC-3′, (antisense): 5′-GTACTCCTGGTCTTCAATGTT-3′ Rodent-iNOS (sense): 5′-AAGTACGAGTGGTTCCAGGA-3′, (antisense): 5′GCACAGCTGCATTGATCTCG-3′ Rodent-p53 (sense): 5′-GGACGACAGGCAGACTTTTC-3′, (antisense): 5′-GGCACAAACACGAACCTCAAA-3′ Rodent-Bax (sense): 5′-ATGGCTGGGGAGACACCTGA-3′, (antisense): 5′-GCAAAGTAGAAGAGGGCAACC-3′ Rodent-Bcl-2 (sense): 5′-CCTTCTTTGAGTTCGGTG-3′, (antisense): 5′-GAGACAGCCAGGAGAAT-3′ Rodent- STAT1 (sense): 5′-CATGGAAATCAGACAGTACCT-3′, (antisense): 5′-TCTGTACGGGATCTTCTTGGA-3′.

### siRNA sequences

The rodent set of siRNAs was designed based on the homologous sequences in the rat and mouse cDNA sequences. Custom siRNA was purchased from Qiagen. Scramble siRNA was also procured from Human/Mouse Starter Kit (Qiagen, Germantown, MD, USA). Rodent STAT1 siRNA target sequence was 5′-AAGGAAAAGCAAGCGTAATCT-3′,^20^ while rodent STAT3 siRNA target sequence was 5′-CTGAGTTGAATTATCAGCTTA-3′.

### siRNA transfection

RNAfectin (Applied Biological Materials Inc., Richmond, BC, Canada) was used for all transfections of siRNAs, respectively, according to the manufacturer’s instructions. Briefly, the UB/OC-1 cells were transfected with 10 nM of STAT1 siRNA, 10 nM of STAT3 siRNA or scramble siRNA in serum-free medium for 8–12 h. The culture medium was then replaced with fresh medium for 48 h. Cells were then treated as indicated.

### Luciferase assay

UB/OC-1 cells were transfected with 1 *μ*g of STAT1 luciferase plasmid and 0.25 *μ*g of *Renilla* plasmid using Lipofectamine 3000 reagent (Thermo Fisher Scientific) according to the manufacturer’s protocol. Cells were pre-treated with EGCG (100 *μ*M) for 45 min, followed by cisplatin (2.5 *μ*M) for 8 h. Luciferase activity was assessed by Dual-Luciferase Reporter Assay Kit (Promega, Madison, WI, USA) according to the manufacturer’s protocol. Briefly, the cells were harvested using the lysis buffer provided with the kit. Aliquots (25 *μ*l) of the lysates were mixed with 25 *μ*l luciferase assay substrate and luciferase activity was measured using a GloMax-Multi Detection System (Promega). Aliquots (25 *μ*l) of Stop and Glo substrate were then added to the lysate mixture to measure the activity of *Renilla* luciferase, which was used for normalization.

### LDH assay

LDH assay was performed using the Pierce LDH Cytotoxicity Assay Kit (Thermo Fisher Scientific) according to the manufacturer’s instructions. Cells (~3500 cells per well) were seeded into a 96-well plate. After respective treatments, cells were allowed to grow for 48 h. Total LDH release (100%) was determined by adding 10 *μ*l of sterile ultrapure water and 10 *μ*l of lysis buffer (× 10) to another set of the wells containing cells. For UB/OC-1 cells, the plates were incubated at 33 °C in 10% CO_2_ for 45 min. Fifty microliters of each sample medium were transferred to a 96-well flat bottom plate. Reaction mixture of 50 *μ*l was added to each sample well and mixed by gentle tapping. The plate was further incubated for 45 min, and absorbance was recorded at 490 and 680 nm using an ELISA plate reader (EL800 Universal Microplate Reader from BioTek Instruments, Winooski, VT, USA). Percent cytotoxicity was estimated as the LDH release in the drug treatment groups relative to the total release produced by lysis buffer and was calculated based on the manufacturer’s instructions.

### MTS assay

Cell viability was performed by using CellTiter 96 AQueous One Solution Cell Proliferation Assay Kit (Promega), according to the manufacturer’s instructions. In brief, 3500 UB/OC-1 cells per well were seeded into a 96-well plate. After respective treatments, cells were allowed to grow for 48 h. After 48 h, 20 *μ*l of CellTiter 96 AQueous One Solution (Promega, Madison, WI, USA) reagent was added to each well in 100 *μ*l of total volume of media. Cells were incubated for 1 to 2 h, and absorbance was recorded at 490 nm using an ELISA plate reader. The absorbance is directly proportional to the number of living cells and is expressed as a percent of vehicle-treated cells.

### CellROX assay (ROS assay)

ROS generation was measured with CellROX green reagent (Thermo Fisher Scientific). Briefly, UB/OC-1 and UMSCC 10B cells were pretreated with EGCG (100 *μ*M) for 45 min, followed by cisplatin (2.5 *μ*M) treatment for 45 min. Then, UB/OC-1 and UMSCC 10B cells were treated with 5 *μ*M CellROX green reagent and incubated at 33 °C and 37 °C for 30 min, respectively. Next, cells were washed gently with PBS three times and fixed with 3.7% formaldehyde for 15 min. UB/OC-1 and UMSCC 10B cells were then treated with Hoechst (1 : 2000 dilution, 10 mg/ml stock) treatment for 20–30 min in the dark. Next, cell samples were mounted on slides using ProLong gold and imaged using a Leica Laser Scanning Confocal Microscope. Image analysis was performed using the Leica Software version LAS-AF-Lite-2.6.0-266.

### Apoptosis detection by flow cytometry

The apoptosis assay was performed using Annexin V-FITC and PI Apoptosis Detection Kit (BD Pharmingen, San Diego, CA, USA) according to the manufacturer’s instructions. At the end of the treatment, cells were washed with PBS and harvested in a 0.5% trypsin/EDTA solution at 37 °C, centrifuged at 220x*g* for 5 min and then immediately resuspended in the buffer provided in the kit. Cells (~1 × 10^5^ cells/500 *μ*l) were then maintained in the dark for 15 min at room temperature with 5 μl of both FITC-conjugated Annexin V and propidium iodide. Samples were analyzed immediately by a flow cytometer (BD FACSCalibur from BD Biosciences, San Jose, CA, USA). The results were analyzed using the BD CellQuest Pro (BD Biosciences, San Jose, CA, USA) Software. Apoptosis was determined by adding the cell populations displayed in the lower and upper right-hand quadrant of the dot plot, which indicates cells in early apoptosis and necrosis and late apoptosis phases, respectively.

### Western blot analysis

Western immunoblotting was performed as described previously.^16^ Following treatments, the cells were washed once with ice-cold 1 × PBS and whole-cell lysates were prepared by homogenizing in ice-cold lysis buffer containing 50 mM Tris-HCl, 10 mM MgCl_2_ and 1 mM EDTA in the presence of protease inhibitor (Sigma-Aldrich) and phosphatase inhibitors 2 and 3 (Sigma-Aldrich). Total cellular proteins were then resolved on acrylamide gel and transferred onto the nitrocellulose membrane and probed with specific primary antibody. Blots were then incubated with species-specific fluorescent-tagged IgG secondary antibody and scanned and visualized using LI-COR Odyssey Imaging System (LI-COR Biosciences). Densitometric analysis of the bands was performed using the LI-COR Odyssey 3.0 Software (LI-COR Biosciences, Lincoln, NE, USA).

### Protein determination

The level of protein in samples was determined by the Bradford method,^74^ using bovine serum albumin to prepare standard curves.

### Statistical analysis

Statistical significance differences among groups were performed using Student’s *t*-tests or analysis of variance with Tukey’s multiple comparisons test correction using the Prism 6.07 software (GraphPad Prism, La Jolla, CA, USA) Errors bars shown in the figures and data presented in the text represent the mean±S.E.M., with *n* (number of individual experiments)≥3.
